# Antibody Responses to SARS-CoV-2 at 8 Weeks Postinfection in Asymptomatic Patients

**DOI:** 10.3201/eid2610.202211

**Published:** 2020-10

**Authors:** Pyoeng Gyun Choe, Chang Kyung Kang, Hyeon Jeong Suh, Jongtak Jung, EunKyo Kang, Sun Young Lee, Kyoung-Ho Song, Hong Bin Kim, Nam Joong Kim, Wan Beom Park, Eu Suk Kim, Myoung-don Oh

**Affiliations:** Seoul National University Hospital, Seoul, South Korea (P.G. Choe, C.K. Kang, H.J. Suh, E.K. Kang, S.Y. Lee, N.J. Kim, W.B. Park, M.-D. Oh);; Seoul National University Bundang Hospital, Seongnam, South Korea (J. Jung, K.-H. Song, H.B. Kim, E.S. Kim)

**Keywords:** respiratory infections, severe acute respiratory syndrome coronavirus 2, SARS-CoV-2, SARS, COVID-19, 2019 novel coronavirus disease, coronavirus disease, zoonoses, viruses, coronavirus, asymptomatic, neutralizing antibody

## Abstract

We compared levels of severe acute respiratory syndrome coronavirus 2 neutralizing antibodies in recovery plasma from 7 completely asymptomatic coronavirus disease patients with those in symptomatic patients in South Korea. We found that serologic diagnostic testing was positive for 71% (5/7) of completely asymptomatic patients, but neutralizing antibody response occurred in all 7 patients.

Severe acute respiratory syndrome coronavirus 2 (SARS-CoV-2), a new strain of betacoronavirus that causes coronavirus disease (COVID-19), quickly spread worldwide; the World Health Organization declared COVID-19 a pandemic on March 11, 2020 (*1*). Recent studies showed that a substantial number of asymptomatic COVID-19 patients contributed to the rapid dissemination of SARS-CoV-2 (*2*). In hospitalized COVID-19 patients, neutralizing antibody production was shown to increase after the first week of symptom onset, which correlated with disease severity (*3*,*4*). However, the neutralizing antibody response in asymptomatic patients is unclear.

In this study, we analyzed the completely asymptomatic COVID-19 patients who were isolated in a community treatment center (CTC) operated by Seoul National University (SNU) Hospital in response to a huge COVID-19 outbreak in Deagu, South Korea. During the CTC stay, physicians and nurses comprehensively evaluated the patients using a video consultation system twice daily (*5**–**7*). The completely asymptomatic patients were defined as those with body temperature <37.5°C and no symptoms (e.g., subjective fever, myalgia, rhinorrhea, sore throat, cough, sputum, chest discomfort) during the entire CTC stay. A total of 15 completely asymptomatic patients were confirmed among 113 patients with SARS-CoV-2 infection in the CTC (*8*). We also evaluated COVID-19 patients with pneumonia who were admitted to the Biocontainment Unit in SNU Hospital and SNU Bundang Hospital (Seongnam, South Korea). We classified pneumonia cases as subtle pneumonia (infiltrations were observed only in the computed tomography images) or apparent pneumonia (infiltrations were observed in chest radiograph) with mild or severe manifestation; case-patients with severe pneumonia required oxygen therapy.

We semiquantitatively measured SARS-CoV-2 IgG using a commercial ELISA kit (Euroimmun, https://www.euroimmun.com) according to the manufacturer’s instructions. Optical density ratio (sample/calibrator) was interpreted as positive (>1.1), borderline (>0.8 to <1.1), or negative (<0.8) according to the manufacturer’s recommendation. We performed neutralization assays as previously described (*9*), using the BetaCoV/Korea/SNU01/2020 virus (*10*) and 2-fold serially diluted plasma samples (2-fold to 4,096-fold). We recorded the highest dilution of plasma that showed inhibition activity of SARS-CoV-2 as the neutralizing antibody titer. We performed the assay in duplicate with negative control samples from healthy volunteers and patients 7–12 months after recovery from laboratory-confirmed Middle East respiratory syndrome coronavirus infection. The Institutional Review Boards of Seoul National University Hospital approved the study (IRB no. H-2004-158-1118).

Seven completely asymptomatic COVID-19 patients from the CTC and 17 patients with COVID-19 pneumonia from SNU-affiliated hospitals participated in this study (Appendix Table). Of the completely asymptomatic patients, ELISA showed positive results in 5 (71%) patients, borderline result in 1 (14%) patient, and negative result in 1 (14%) patient. ELISA showed higher optical density value in patients with pneumonia; titers correlated with disease severity (Figure). All patients showed neutralizing antibody response. We calculated the geometric mean titer of neutralizing antibody in all asymptomatic patients and in 4 of each type of pneumonia patient (subtle, mild, or severe); geometric mean titer was 78 in asymptomatic patients (n = 7), 256 in patients with subtle pneumonia (n = 4), and 3,158 in patients with apparent pneumonia (n = 8; 4 mild and 4 severe cases).

**Figure:**
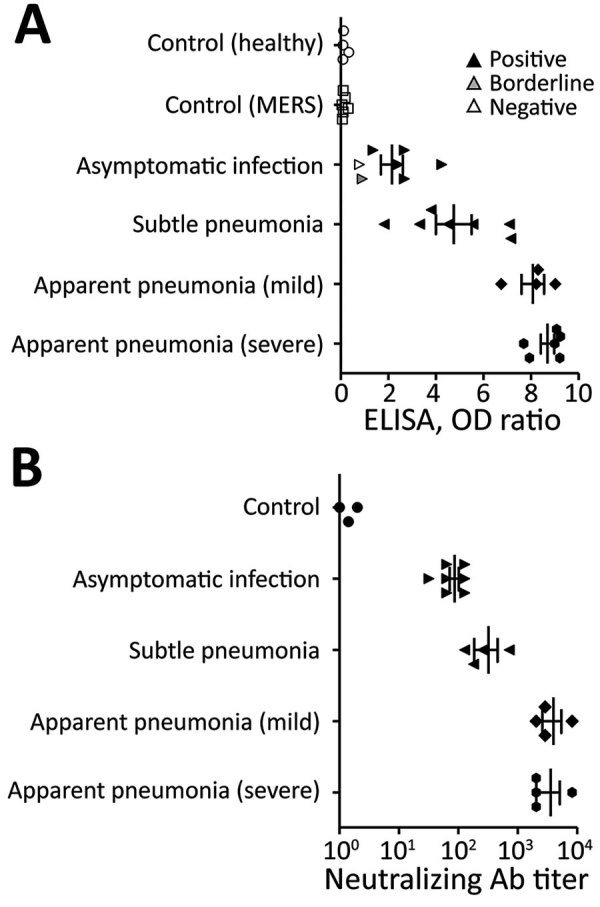
Antibody response against severe acute respiratory syndrome coronavirus 2 at 8 weeks postinfection among patients and controls in South Korea. A) Serologic diagnostic test (ELISA) results. OD ratio indicates the ratio of the extinction of the patient sample over the extinction of the calibrator. B) Neutralization assay results. For each patient type, an outlined symbol indicates a negative test result, gray symbol a borderline result, and black symbol a positive result, as tested according to manufacturer recommendation. Bars represent mean values and SE. From each patient group other than the completely asymptomatic group, 3–4 patients were randomly selected for neutralization assay. The controls included 1 healthy volunteer and 2 patients with MERS. Ab, antibody; MERS, Middle East respiratory syndrome; OD, optical density.

Neutralizing antibodies play an essential role in virus clearance and have been considered a critical immune player for protection against viral diseases. Knowledge of the neutralizing antibody response in asymptomatic patients is critical for diagnosing the disease, understanding pathogenesis, and interpreting seroepidemiologic data to define prevalence and risk factors for infection. Production of neutralizing antibodies in asymptomatic COVID-19 patients was reported recently. Wu et al. reported that ≈30% of recovered mild COVID-19 patients generated a deficient level of neutralizing antibody titers; in 10 of the 175 patients, the level was below the limit of detection (F. Wu et al., unpub. data, https://doi.org/10.1101/2020.03.30.20047365). The difference in results from our study compared with the previous study might be caused by differences in the timing of the test. In the previous study, antibody tests were performed 2–3 weeks after symptom onset, whereas we tested 2 months after symptom onset or laboratory diagnosis. Seroconversion in asymptomatic patients might take longer.

In our study, the neutralizing antibody titer correlated with the severity of the disease. This result suggests that patients with more severe disease might be more protected against reinfection and those with asymptomatic or mild disease could be more vulnerable to waning immunity over time because the initial immune response was not as strong as in patients with more severe disease.

The ELISA results showed good agreement with the neutralizing antibody results. Negative ELISA results in some asymptomatic patients may be a limitation of the ELISA or may be attributed to patients with cross-neutralizing antibodies in their serum. Despite the limitation of our small sample size, our findings suggest that seroepidemiologic studies may detect mild COVID-19 infection in completely asymptomatic patients by the presence of neutralizing antibodies at 8 weeks postinfection. 

Appendix Additional information about antibody response to SARS-CoV2 at 8 weeks postinfection.
