# Supplementary material for: Antibody Responses to SARS-CoV-2 at 8 Weeks Postinfection in Asymptomatic Patients
Source: Emerg Infect Dis. 2020 Oct;26(10):2484–7. doi: 10.3201/eid2610.202211 (PMC7510710; doi:10.3201/eid2610.202211)
Supplement: Appendix — Additional information about antibody response to SARS-CoV2 at 8 weeks postinfection. [file 20-2211-Techapp-s1.pdf]

Article DOI: https://doi.org/10.3201/eid2610.202211

Antibody Responses to SARS-CoV-2 at 8 Weeks Postinfection in Asymptomatic Patients

Appendix

Appendix Table. Clinical characteristics of patients in study of antibody responses to severe acute respiratory syndrome coronavirus 2 at 8 weeks postinfection

Characteristics	Completely asymptomatic	Subtle pneumonia	Apparent pneumonia, mild	Apparent pneumonia, severe
No. patients	7	7	4	6
Male sex, n (%)	5 (72)	3 (43)	3 (75)	5 (83)
Age, median y (range)	25 (20–28)	53 (24–84)	59 (43–78)	75 (55–86)
Isolation period*, median no. days (range)	29 (21–36)	18 (11–21)	17 (11–18)	28 (15–77)
Symptom onset to antibody testing, median no. days (range)	55 (55–60)†	62 (46–66)	45 (39–68)	51 (44–75)

*Patients were deisolated when their SARS-CoV-2 rRT-PCR test results from 2 tests performed with a 24-h interval were negative.
†Duration from laboratory diagnosis to antibody testing in asymptomatic patients. Of the asymptomatic patients, 2 patients had an exposure history.
